# Parental Home Vision Testing of Children During Covid-19 Pandemic

**DOI:** 10.22599/bioj.157

**Published:** 2021-01-21

**Authors:** Sally Painter, Laura Ramm, Laura Wadlow, Maria O’Connor, Bavnesh Sond

**Affiliations:** 1 Birmingham Children’s Hospital, GB

**Keywords:** paediatrics, vision testing, smartphone applications, amblyopia

## Abstract

**Background:**

The Covid-19 pandemic necessitated social distancing restrictions, which placed limitations on access to ophthalmic care to only those who had an imminent risk of sight loss. All other face-to-face consultations were converted to telephone consultations or were postponed. We investigated whether parents were able to test their child’s vision using available home vision testing applications, with an aim to aid decision making during a telephone consultation.

**Methods:**

Families with follow-up consultations at Birmingham Children’s Hospital were asked to test their child’s vision at home. Instructions for the use of Peek acuity, or iSight Pro, were emailed to a parent. Parents chose to use a particular app based on available devices at home. Parents were asked to test uniocular visual acuity twice. Home versus hospital acuity was correlated. Home acuity test-retest reliability was acquired. Parental feedback was obtained through questionnaires.

**Results:**

One hundred and three families were contacted, 15 families completed home vision testing. Ten families used Peek acuity, five families used iSight Pro. Uniocular visual acuity test-retest reliability was 0.03 LogMAR. Home-hospital acuity testing had a bias of 0.14 LogMAR, hospital acuity yielding a lower LogMAR score. Most families who completed testing found it easy to do; however, some struggled, and 81 families did not undertake home vision testing.

**Conclusions:**

Uptake of home vision testing was limited by parental engagement, most likely influenced by the current pandemic. Most families who undertook home vision testing were able to generate results that could be used for clinical decision making. Extending the impact of parental vision testing will require education by clinicians and further study to increase sample sizes and to improve confidence.

## INTRODUCTION

The United Kingdom entered lockdown to prevent the spread of SARS- CoV 2 (Covid-19) on 23 March 2020 (Office of the Prime Minister 2020). Restrictions on access to healthcare services were implemented. Face-to-face hospital consultations were limited to those who had sight-threatening conditions or who were at imminent risk of losing sight. Local optometry and community-based orthoptic appointments were also suspended. All patients entering healthcare services were triaged according to clinical need and risk of imminent sight loss (RCOphth 2020a). Those patients who were unable to attend for a face-to-face appointment were offered a telephone consultation or their appointment was postponed.

In the first four weeks of lockdown, Birmingham Children’s Hospital eye department had an average of 45 face-to-face consultations per week, compared to a usual working week where 490 consultations would take place. In contrast, 330 telephone consultations were undertaken.

In a face-to-face consultation, every patient entering the eye department receives a visual acuity test. Telephone consultations have been shown to be satisfactory to patients and clinicians alike, particularly when patients need to travel long distances to see a clinician (Cusack & Taylor 2010). Whilst telephone consultations are beneficial for enquiring about symptoms and medications and discussing concerns with patients and their carers, one limitation is the inability to test vision, unless the patient is with a healthcare provider in another centre (Rathi et al. 2017; Host, Turner & Muir 2018). Visual acuity assessment over the telephone is limited to subjective information from a verbal child or a carer or very crude qualitative observations of vision-related behaviour.

If objective quantitative evidence of visual acuity could be obtained, clinicians would be able to improve triage of urgent cases; monitor visual acuity where this measurement is critical for patient care; enable management decisions to be made remotely and, importantly, reduce footfall into the eye department at a time when this is critical to patient and staff safety.

Printed visual acuity charts can be downloaded by families, and visual acuity information can be reported back to the clinician during the time of the phone call. Limitations to this approach include the need to access a printer and limited optotype size availability (ABCD 2020).

Home vision testing applications (apps) exist and have been validated for use by healthcare professionals (Bastawrous et al. 2015; Milling et al. 2015; Zhao, Stinnett & Prakalapakorn 2019). They are not used in our routine clinical practice, by parents, or carers. Guidance issued by the Royal College of Ophthalmologists (RCOphth) and British and Irish Orthoptic Society (BIOS) suggested caution and interpretation of the results under strict guidance by healthcare professionals (RCOphth 2020b).

At the onset of lockdown, we chose two home vision testing apps that were accessible, free to download and use, were validated and carried CE marks, and used optotypes that matched our clinical practice: iSight Pro and Peek (Kay Pictures 2020; Peek Vision 2020). iSight Pro (Kay Pictures Ltd, Tring, UK) is available to Apple users (Kay Pictures 2020). It uses standard letters or Kay Picture optotypes to allow testing of both near and distance vision. Peek acuity (Peek Vision Ltd, Berkhamstead, UK) is available to Android device users (Peek Vision 2020). It uses the tumbling E optotype to test distance vision in both verbal and non-verbal children and adults.

## METHODS

Permission for this evaluation was granted by the research department at Birmingham Children’s Hospital. This prospective evaluation aimed to assess the potential for home vision testing using available home vision testing apps iSight Pro and Peek acuity.

Instruction leaflets were generated for each app to enable families to understand how to use the apps correctly (Appendix 1). The instructions were temporarily placed on our trust internet page for the duration of the evaluation, for families to access at home. The instructions reminded families to ensure that the required test distance was measured and the child’s eye was adequately occluded during testing.

Eligible families had to be able to understand written instructions in English and have access to a smart phone or tablet. The child had to be able to perform optotype-based visual acuity assessments. All children were attending for follow-up appointments and therefore had a documented visual acuity measurement from a previous hospital visit for comparison. New patients, and those deemed unable to perform a subjective vision assessment due to age or other limiting factor, were excluded.

Families were contacted via telephone to inform them of their upcoming consultation and were asked to consider undertaking home vision testing prior to their appointment. Instructions for home vision testing were sent via email to a parent and could be accessed on the hospital website. Families chose to use either iSight Pro or Peek acuity based on the electronic devices available at home. Home vision testing results were reported to their clinician, either through a telephone consultation, email or when the family attended for their face-to-face consultation.

Families were asked to complete acuity tests with both eyes open (BEO) first, followed by uniocular acuities, whilst wearing their usual glasses prescription. Families chose which eye to test first. Each family was asked to repeat the series of tests on a separate occasion to assess for test-retest reliability. We compared the parental home vision result to that performed by an orthoptist at their face-to-face hospital consultation or recent hospital visit within three months. Those patients who had not been seen in the hospital for three months had stable conditions, with stable visual acuities.

We assessed parent experience through a questionnaire (Appendix 2). Parents returned the questionnaire via email. Those families who did not return a visual acuity test result were contacted via telephone for feedback to enable understanding of the limitations for completing home visual acuity assessments. Families who did not receive the initial email were guided to the hospital website to access the instructions and offered to complete the test.

Test-retest reliability was assessed using repeatability coefficient (Vaz et al. 2013). The repeatability coefficient generates the meaningful real difference between two tests, giving a value in the units of the original test, in this case LogMAR. Home versus hospital acquired vision was assessed using Bland-Altman analysis. All statistical analysis was performed using IBM SPSS Statistics V26.

A record was kept of all children who used an app and whether a clinical decision was based on the information provided. The clinician treating the child was asked to use their own judgment before making clinical decisions based on home test results.

## RESULTS

**Figure 1:**
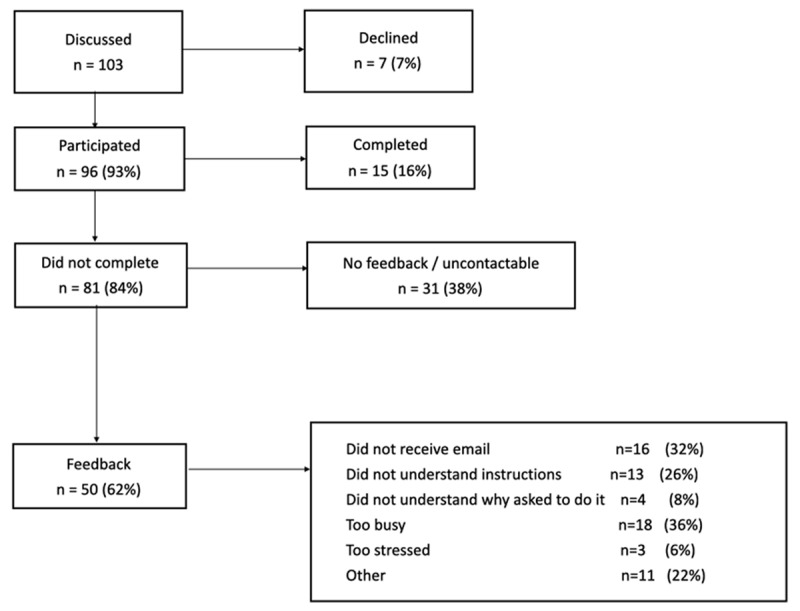
Flow chart of patient involvement.

Over an eight-week period beginning on 14 April 2020, 103 families were contacted to consider home vision testing. Seven families declined at the initial contact. Ninety-six families participated in the home vision testing trial, of whom 15 families provided uniocular home vision tests for each eye of their child (***Figure 1***). The median age of the children participating was seven years old (range 4–15).

### 1. PATIENT ENGAGEMENT

Of 96 families who agreed to participate, 81 (84%) did not provide results. We were able to contact 50/81 who reported a failure to receive the email with instructions (n = 16, 32%), an inability to understand the instructions (n = 13, 26%), too busy to undertake the test (n = 18, 36%), too stressed to complete the test (n = 3, 6%), didn’t understand why they were asked to do it (n = 4, 8%), or other reasons (n = 11, 22%), which included an unwillingness to download an app, no access to a suitable device, or an unwillingness to share data with an unknown company.

The 16 families who failed to receive the email were given verbal instructions on how to access the instructions via the hospital intranet. None of the 16 families completed the vision test.

### 2. EASE OF USE OF APPLICATION

Fifteen families completed home vision testing, 12/15 families completed the questionnaire. Ten families used Peek, five families used iSight Pro. The choice of device was determined by the family and the available devices at home. The results of the questionnaire are seen in ***Table 1***.

**Table 1:** Parental experience of performing home vision testing.


Question:	Parental response (n, %)		

“I found…..”	Easy	No problems	Difficult

**Finding instructions on the website**	8 (75%)	4 (25%)	0

**Understanding the test**	7 (58%)	4 (25%)	1 (8%)

**Testing the child**	4 (33%)	4 (33%)	4 (33%)

**Measuring test distance**	3 (27%)	7 (64%)	1 (9%)

**Keeping the child’s concentration**	4 (33%)	2 (17%)	6 (50%)

**Covering one eye**	3 (25%)	6 (50%)	3 (25%)


### 3. TEST-RETEST RELIABILITY

**Figure 2:**
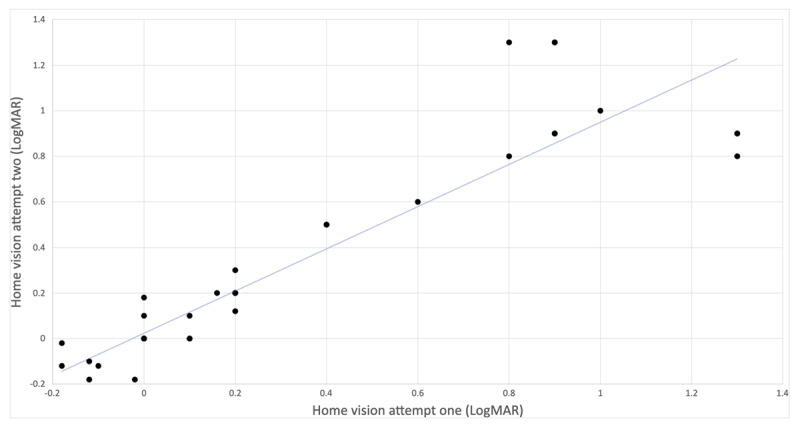
Repeatability of home testing results between first and second attempts.

Thirteen families completed BEO and uniocular tests twice (***Figure 2***). BEO and uniocular results were considered as individual data points. The repeatability coefficient was 0.03 LogMAR with a 95% CI between –0.08 and 0.04. Overlap of some data points prevents visibility of all individual points.

### 4. HOME VISION TEST COMPARED TO HOSPITAL ACQUIRED ACUITY TEST

**Figure 3:**
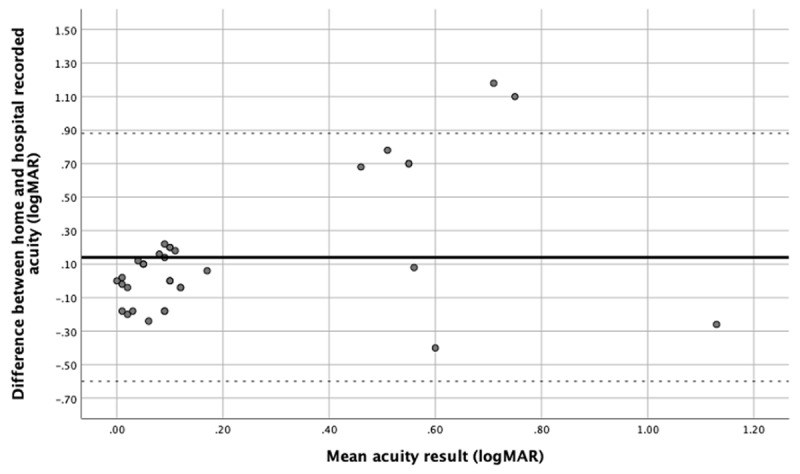
Bland-Altman plot of home visual acuity correlated with hospital visual acuity. Bias = 0.14 LogMAR (solid line), Upper limit of agreement = 0.88 LogMAR (upper dashed line), Lower limit of agreement = –0.60 LogMAR (lower dashed line).

Four families completed home vision testing within five days of attending the hospital. Eleven children did not attend hospital for a face-to-face consultation; therefore, their home vision testing result was compared to the most recent hospital acquired result, all within the preceding three months. Uniocular acuities were considered as individual data points (***Figure 3***). Bias is 0.14 LogMAR; therefore, overall, hospital visual acuity produces a lower LogMAR score (better visual acuity). The upper and lower levels of agreement are 0.88 LogMAR and –0.60 LogMAR, reflecting the wide variation of results between hospital and home acquired test results.

Twenty out of 30 (66%) home testing results fall within 0.2 LogMAR of the hospital acuity result; however there are outliers who fall outside the 95% upper limit of agreement.

One family recorded home vision results that had a difference greater than 0.7 LogMAR from their recorded hospital acuity in each eye. Due to our small sample size, these data points skewed the upper and lower levels of agreement significantly. None of the children reported a subjective change in vision during their consultation.

## DISCUSSION

Birmingham Children’s Hospital is a regional tertiary referral centre for complex ophthalmic conditions and accepts secondary referrals from Birmingham and the Midlands. Birmingham is the UKs second largest city, with a population of approximately 1.1 million. It is a culturally diverse city, and it is estimated that a quarter of residents were born overseas (Office of National Statistics 2011). Over 4 in 10 residents are from an ethnic group other than White British (Birmingham Child Poverty Commission 2016). Over one third of the city’s children and young people are living in poverty, the second highest child poverty rate across the UK’s major cities (Birmingham Child Poverty Commission 2016). It is therefore possible that digital poverty may have impacted the accessibility of this study to our population.

Implementation of home vision testing by parents was the biggest challenge faced by our team. Of 103 families contacted, 7 declined at the outset, and a further 81 did not complete the test. Only 14.6% of families approached were able to complete the test and provide a test result. Of the families that completed the test, nearly half had parents who volunteered the information that a healthcare professional in the household had helped facilitate the test. Due to the small number of children who completed testing, we are unable to separate the experiences of users of each app.

At the time of commencing the study, the UK was in lockdown. All adults were asked to work from home if possible or were furloughed from their current jobs. All schools were closed and parents were asked to undertake home schooling of their children. We were unable to contact 31 families, most likely due to multiple demands placed on the families at this time. Twenty-one out of 50 families were either too busy or too stressed to undertake the testing of their children. It is possible that asking families to undertake this study at a different period of time may have yielded greater engagement and participation. It is also possible that if more parents were at work and children were attending school, uptake may have been less than we experienced.

Thirteen families did not understand the instructions for use of the application. Both written and pictorial information was included, and the literature contained video tutorials on how to use the applications. We would like to explore the reasons for not understanding the instructions in order to be able to improve their accessibility. Some families did not wish to download an application that could potentially collect personal information. If home vision testing is to be used in future clinical practice, parent education and education on the use of the application would be necessary to ensure that all families could access the technology. Reassurance on information security would also need to be provided.

Fifteen families provided visual acuity data for their child. From the feedback provided, they found the apps easy to use and found it easy to measure out a test distance. However, we acknowledge that there was no way of monitoring if the apps were used correctly or if the correct testing distance was measured. Parents found it difficult to ensure that their child continued to concentrate throughout the test and to ensure that one eye was occluded throughout. Orthoptists are particularly skilled and experienced at judging this and know how to encourage children to achieve their best possible visual acuity measurement; skills we cannot expect parents to have instinctively. The parents who found it difficult were able to generate accurate results; there was no agreement between the inaccurate test results and ease of use of the applications.

Thirteen children provided repeat data sets that showed a small coefficient of repeatability.

Four children had an inter-test variation of >0.3LogMAR. Whilst the majority of families were able to achieve accurate test repeatability, it was not possible for all families to do this. We acknowledge that our data is from a small sample size and that larger numbers of patients would be required in order to improve confidence in home testing applications. In the original paper describing Kay Picture optotypes, the mean bias was 0.01LogMAR; in our study the coefficient of repeatability was 0.03LogMAR (Milling et al. 2015).

By the nature of parent-led home vision testing, it is not possible to control visual acuity measurement, control the device used for the test, or control the quality of the image generated by the screen. Test distance accuracy and optotype measurement at the beginning of the test are reliant on the parent checking them prior to testing vision. The applications are also limited by the size of the screen. Peek advises you to move the screen closer to the child if the child cannot see the largest optotype. iSight has two testing distances—3m and 1.5m—which enable the optotypes to be enlarged. The child peeking through fingers or sneaking towards the device needs to be noticed by the parent, and the parent needs to implement a change. An orthoptist would encourage a child to see one more line when they decline to read any more, but it is unknown whether a parent would do the same.

Whilst most of our patients acquired a visual acuity at home that was comparable to hospital acuity results, we had outliers who fell outside of the normal distribution for the difference between tests. Upon closer analysis of the data, over half of the outlying data points were provided by one family for two different children. This could indicate that they did not perform the test as instructed or that it was reported incorrectly. These children recorded very low visual acuities with home vision testing. They had previously recorded normal visual acuity levels and had no subjective visual deterioration. By comparing the home vision testing result with the last recorded hospital measured visual acuity and analysing it with the subjective assessment of the child’s vision and the known clinical background, it may be possible to determine whether the home vision test result is likely to be accurate. However we are aware that this must be confirmed by a clinician in order to see if there has been any deterioration in visual acuity. Given the discordance between history and reported vision assessments, the outlying home acuity result was not deemed to be clinically reliable.

Smaller differences between hospital and home visual acuity tests are harder to interpret. It is possible that there is a true deterioration in visual acuity that may not be noticed by the child and therefore not reported in a subjective assessment. It is also possible that there is inter-test variation and the visual acuities are the same as previously recorded. Larger numbers of patients would be required in order to increase the confidence in the results of the home acuity test.

Kay pictures and tumbling Es have been validated against standard LogMAR tests (Shah et al. 2012; Treacy et al. 2015). It is well known that there is variation in test results between single optotypes and linear optotypes. It is also known that there is a potential learning curve when any new test is introduced. We do not routinely use tumbling E in our orthoptic practice and therefore this was a novel test for each patient. There are therefore multiple potential sources of variation in test results.

## CONCLUSIONS

We began this evaluation at the onset of a pandemic and acknowledge we were giving applications, designed for medical professionals, to parents, with limited supporting educational resources. We have shown that the principle of parent-led home vision testing with iSight Pro and Peek acuity is possible and parents can provide visual acuity results. Some families found it easy to obtain results and could produce information that was consistent with their child’s hospital recorded visual acuity. However, some families struggled or were unable to achieve accurate results.

We understand the limitations of analysing data, and drawing conclusions, from a small cohort of children. Studies with larger numbers of children over a greater range of ages and visual ability would be necessary to provide confidence in test-retest reliability and comparison with standard hospital-led vision testing.

The uptake by parents was limited, which may have been influenced by the unique circumstances families were in during lockdown. We suggest that uptake and reliability of home vision testing could be improved by clinician-parent tuition. Initial test results could be taken in hospital to allow direct comparison between home vision app test results and hospital acquired app test results. Patient selection would be critical to ensure engagement from the family and the child.

Optimising parental experience of these technologies is an important focus for future research and should be used as the basis on which to develop apps specifically for parental use.

In some circumstances, clinician-led home vision testing over a video conferencing platform may be appropriate. This would allow more control over the test, such as testing distance, correct occlusion of each eye and allowing a clinician to observe patient behaviour during testing.

This is a rapidly changing field in which we have shown that there is scope for monitoring of visual acuity by a family at home.

## ADDITIONAL FILES

The additional files for this article can be found as follows:

10.22599/bioj.157.s1 Appendix 1. Instructions for iSight app and Peek Acuity Pro.

10.22599/bioj.157.s2 Appendix 2. Parental questionnaire.
