# Supplementary material for: Parental Home Vision Testing of Children During Covid-19 Pandemic
Source: Br Ir Orthopt J. 2021 Jan 21;17(1):13–9. doi: 10.22599/bioj.157 (PMC8269789; doi:10.22599/bioj.157)
Supplement: Appendix 1. — Instructions for iSight app and Peek Acuity Pro. [file bioj-17-1-157-s1.zip › s1-bioj-157_painter/iSight-guide1-4-2020a-v1.pdf]

Laura Naomi Wadlow –Birmingham Children's Hospital

Using iSight Pro– For use on Apple Ipads or Iphone

Downloading the App

Please note, different IOS versions and/or having the app already installed on a device may alter the order in which the following screens appear.

Go to App Store

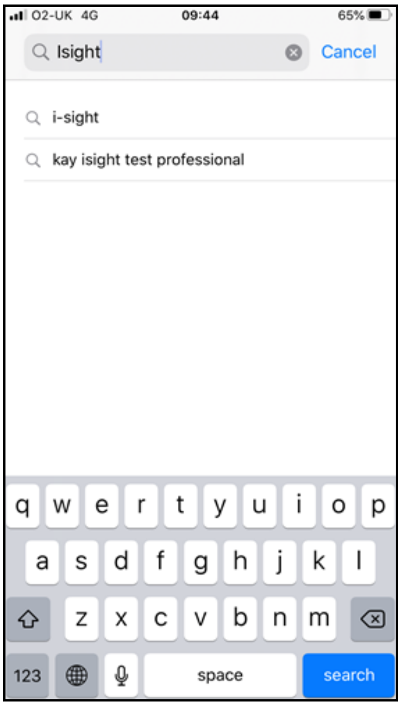

1-Type iSight into search bar

2- Select Kay iSight test professional

3– Click on Get/open/install

4– Sign in with apple ID/fingerprint recognition

5-Click on Open

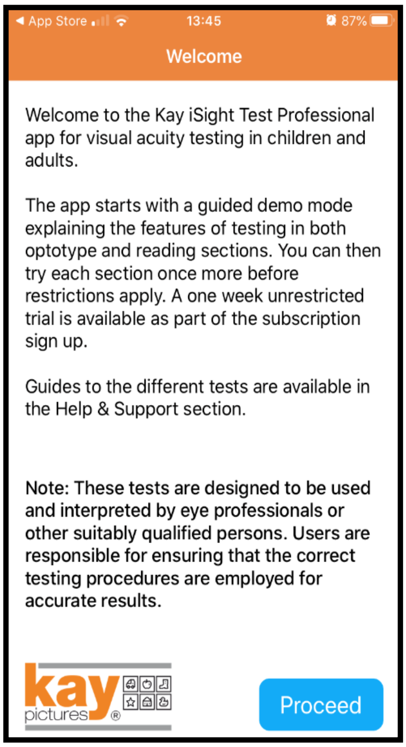

6– Click proceed

Please note that due to COVID –19, the developer is currently offering 6 months FREE subscription! You will not be charged for the demo or for the first 6 months of use!

With thanks to Hazel Kay (Kay pictures) for allowing us to use the iSight app with 6 months free subscription during Covid 19.

Using iSight Pro– For use on Apple Ipads or Iphone

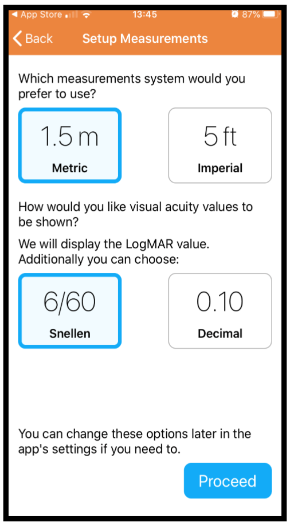

7– Click Proceed

(1.5m metric and 6/60 should be highlighted blue)

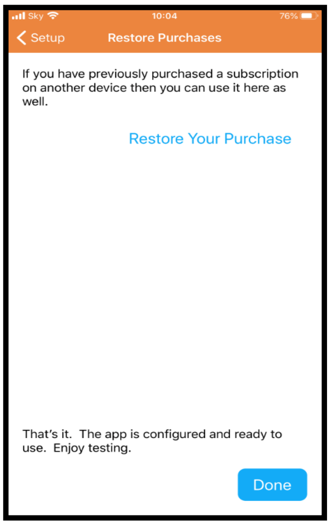

8- Click Done

(or click restore purchase if you have already download-ed it onanother device)

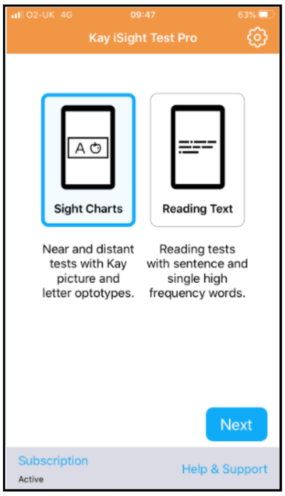

9– Click Next

(sight charts should be high-lighted blue)

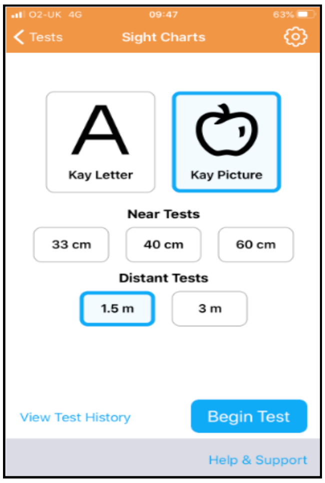

10-Click on Kay picture (apple)

11-Click on 1.5m or 3 m distance test depending on which distance you plan to use

12-Click on Begin test

The app will now take you through a guided demo

Please follow the prompts!

With thanks to Hazel Kay (Kay pictures) for allowing us to use the iSight app with 6 months free subscription during Covid 19.

Using iSight Pro– For use on Apple Ipads or Iphone

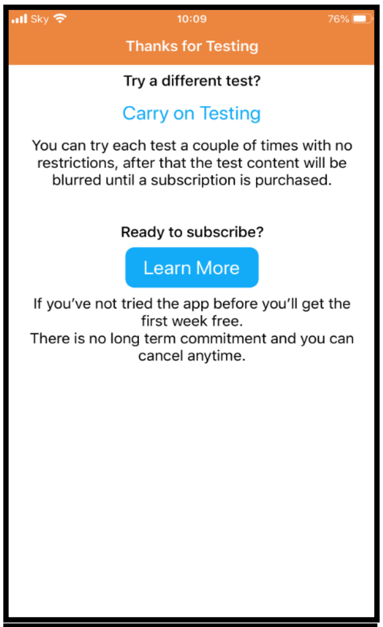

11-Click on Learn more

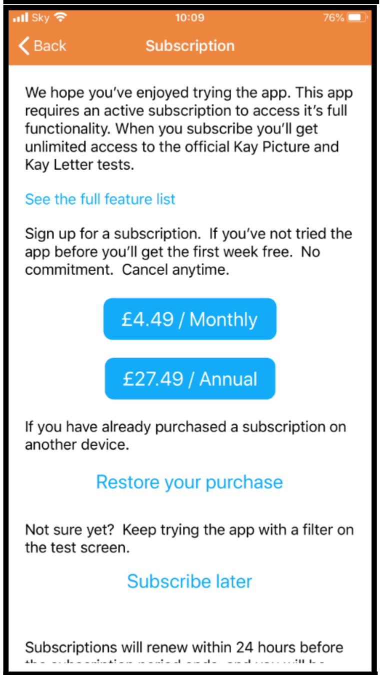

12-Click on £4.49/Monthly

Please note that due to COVID –19, the developer is currently offering 6 months FREE subscription! You will not be charged for the demo or for the first 6 months of use!

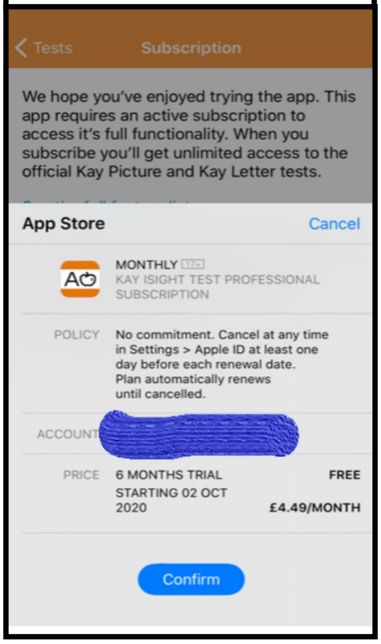

13– Click confirm

Please note the policy on cancellation.

To prevent monthly payments being taken after 6 months you must **cancel your subscription before the end of the free trial**

With thanks to Hazel Kay (Kay pictures) for allowing us to use the iSight app with 6 months free subscription during Covid 19.

Laura Naomi Wadlow –Birmingham Children's Hospital

Using iSight Pro– For use on Apple Ipads or Iphone

Using the App

1-Click on the App icon on your device

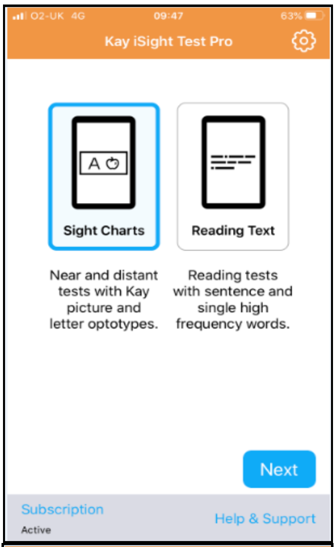

2-Click on Sight Charts

3-Click Next

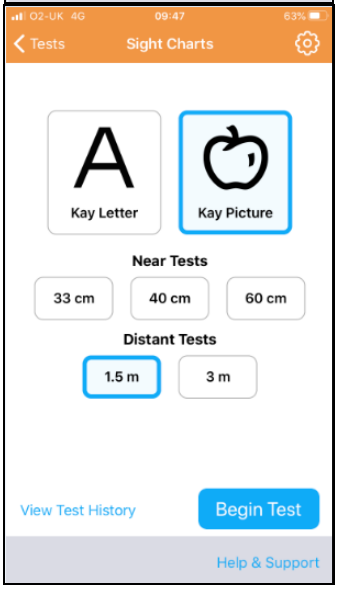

4-Click Kay Picture

5-Click 1.5m/3m distance test –depending on the space you have available

6- Click begin text

Please hold your phone horizontally (landscape)

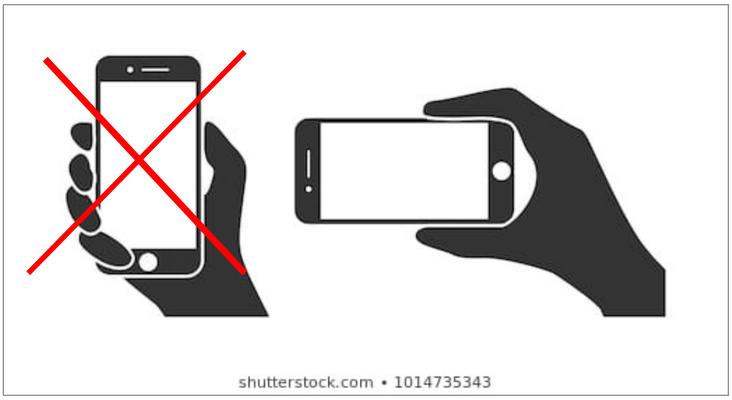

With thanks to Hazel Kay (Kay pictures) for allowing us to use the iSight app with 6 months free subscription during Covid 19.

Laura Naomi Wadlow –Birmingham Children's Hospital

Using iSight Pro– For use on Apple Ipads or Iphone

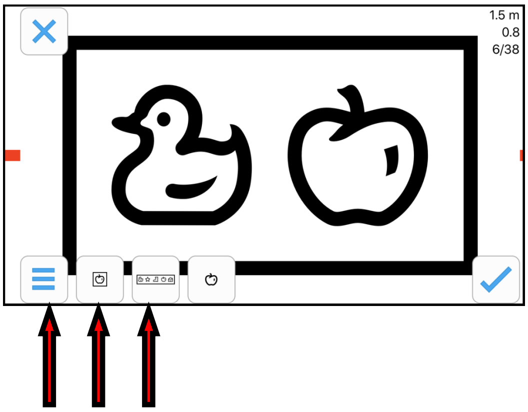

6-Click on the icon 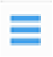

and select 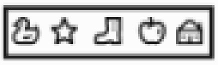 **or** 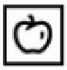

(dependant on childs age/ability)

- **7-Test your child's vision**
- Have your child sit/stand 1.5m or 3m away from the ipad/phone screen, depending on what space you have available and what test distance you chose on the previous screens.
- Hold the screen at eye level
- Cover over the Left eye to test the Right eye, or you may first want to practice with both eyes together in order to check your child understands the test
- Ask your child to name the picture(s)
- If your child can correctly name the picture(s) swipe the screen up to make them smaller
- By swiping left or right you can show more pictures of the **same size**. You will need to do this especially if you are using the single pictures in a box.
- If a child is unable to name the pictures swipe down to make them larger.
- Keep testing until your child can no longer name them all correctly

It is important to make sure your child is not peeping when testing each eye separately. The eye must be covered completely and the child must not be able to see through the cover/patch.

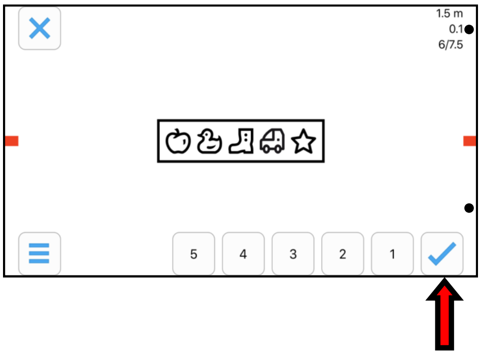

8- Now click on the blue arrow and select how many pictures they were able to name correctly at that size. (maximum of 5)

If a child could not name any pictures at that size you need to check how many they can name correctly on the next largest size (by swiping down)

With thanks to Hazel Kay (Kay pictures) for allowing us to use the iSight app with 6 months free subscription during Covid 19.

Laura Naomi Wadlow –Birmingham Children's Hospital

Using iSight Pro– For use on Apple Ipads or Iphone

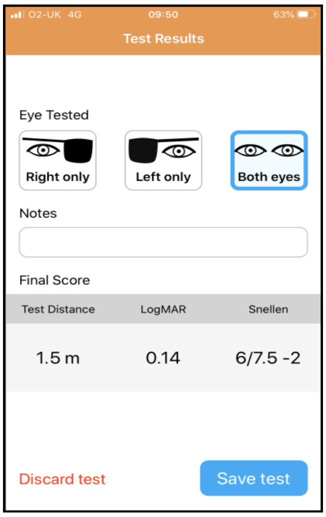

9-Click on Right only, Left only or Both eyes according to which eye(s) you have just tested.

Put any comments in the notes e.g. peeped/leaning forward / disliked left eye being patched/struggled

10 -Click on save test or discard test

Please save the test if you wish to recall the results for your Orthoptist/Ophthalmologist

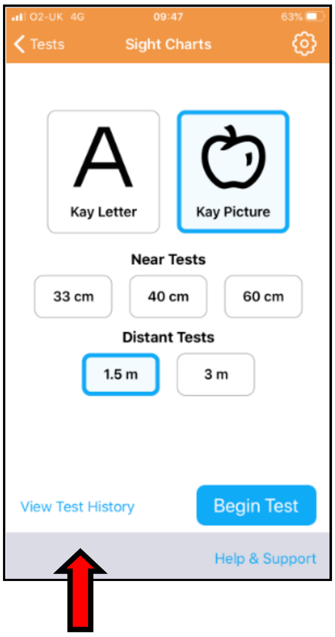

The app will now take you back to this page

Return to step 4 in the guide 'using the app' and follow the instructions again to test the other eye

Click on 'view test history' to view saved tests and to report them to the Orthoptist/ophthalmologist when required

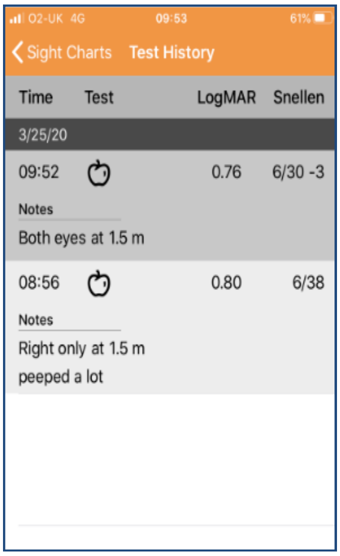

Your eye professional may ask you to take a screenshot of your test history and email it to the eye department

bwc.eyedepartment@nhs.net

Or you may be able to relay the information by telephone:

0121 333 9467 –Eye Department, BCH.

Please call the eye department for assistance.

With thanks to Hazel Kay (Kay pictures) for allowing us to use the iSight app with 6 months free subscription during Covid 19.
