# Supplementary material for: Parental Home Vision Testing of Children During Covid-19 Pandemic
Source: Br Ir Orthopt J. 2021 Jan 21;17(1):13–9. doi: 10.22599/bioj.157 (PMC8269789; doi:10.22599/bioj.157)
Supplement: Appendix 1. — Instructions for iSight app and Peek Acuity Pro. [file bioj-17-1-157-s1.zip › s1-bioj-157_painter/Peek-Acuity-Pro-3.pdf]

Peek Acuity Pro - Home Vision Testing Guide

- This Test uses a **E** shape which points in different directions and changes size to measure eye sight
- This App can be downloaded on Android devices via **Play Store**

1. Search for ‘Peek Acuity Pro’ in Play Store and click ‘Install’

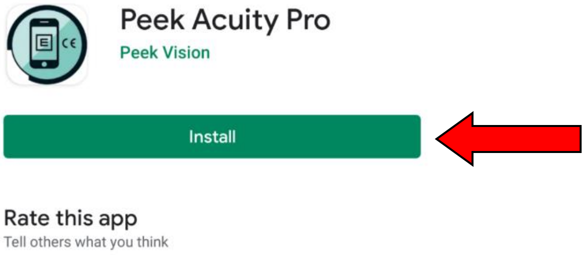

2. A written Tutorial on how to use the app is available on the App Home Page. You can also access a Video Tutorial via YouTube https://www.youtube.com/watch?v=Xw3qMLjdpfM

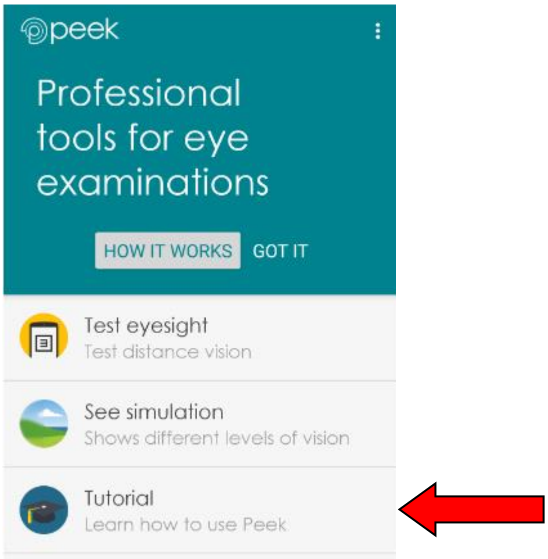

3. Once you have seen the tutorial, return to the App Home Screen and select **‘Test eyesight’**

4. Set the Test Distance as 2m. Do not change the Unit from **‘LogMAR(0.0)**.’ Then select **‘Done’**

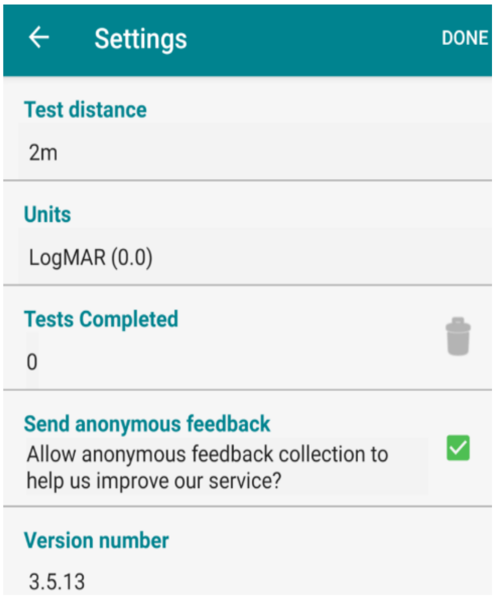

5. You will then need to calibrate the App – see instruction below

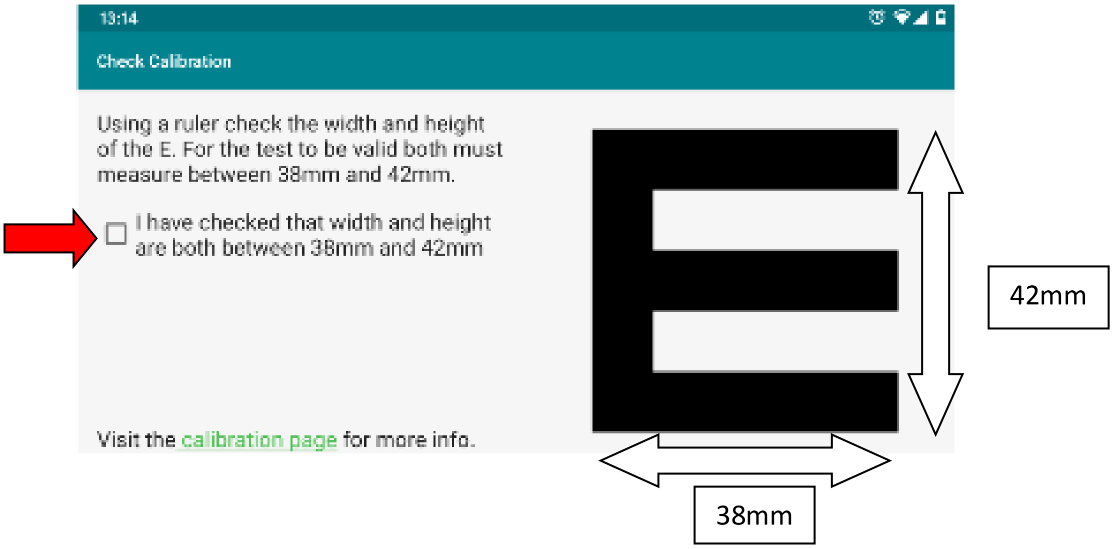

Once you have measured the E and each side measures between 38mm and 42mm it is click on the box to start the Vision Test

6. To perform the test
    - You need to be in a space where it isn't too bright or too dark and that there won't be any sunshine or light bouncing off your device
    - You need to be able to measure the test distances between your device and your child. Measure it on the floor, or have pre-cut pieces of string ready. You need to be able to measure 2m, 1m and 30cm
    - To start, your child needs to sit/stand 2m away from the tablet/phone screen
    - Ensure that you hold the device horizontally (landscape) and at your child's eye level
    - Cover the left or right eye depending on which eye you want to test first. If a hand is used to cover the eye you must ensure the child cannot see. Ideally use something opaque to cover the eye e.g. an eye patch or pirate patch.
    - You may want to practise with both eyes together first in order to determine your child's confidence

7. Ask your child to point in the direction that they think the legs of the E is pointing

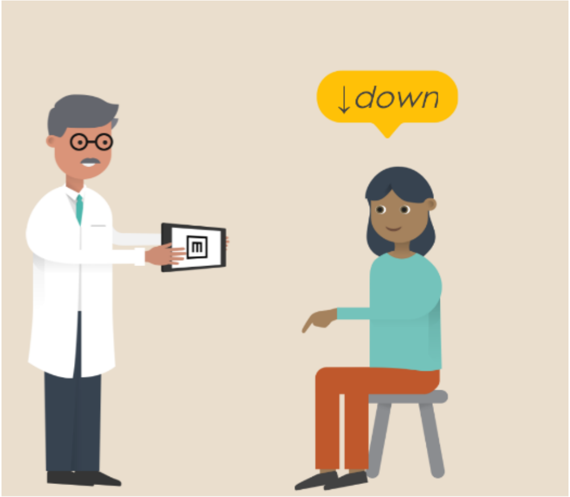

8. Swipe the screen in the direction that your child points. You don't need to check if they are correct

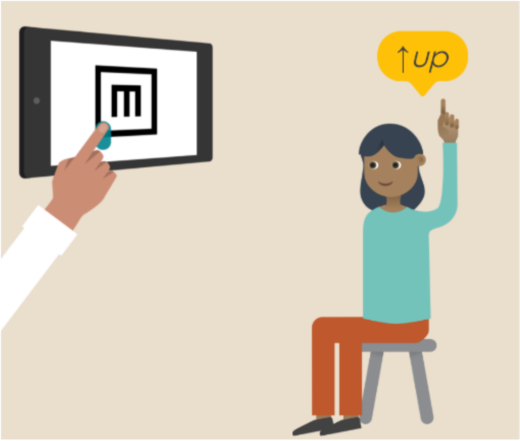

9. If your child is unable to see, **shake the device** and this will change the size and direction of the E

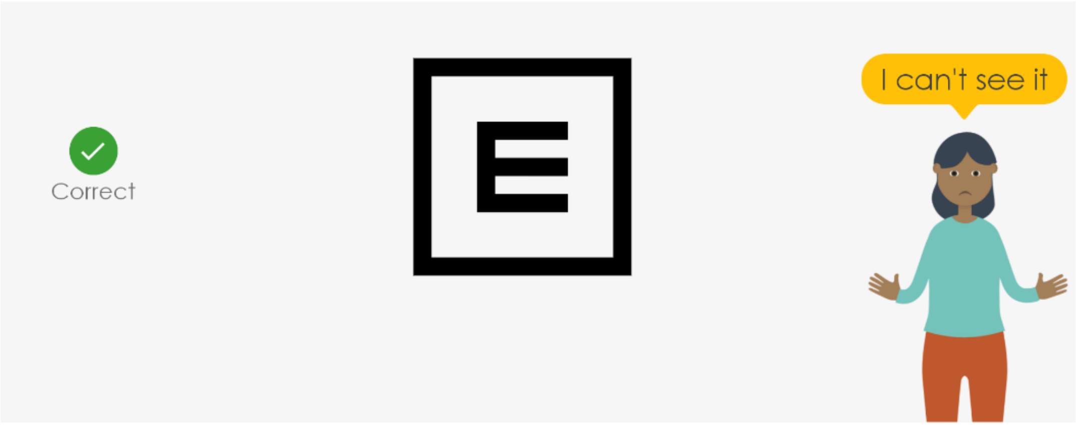

10. If your child is **still unable to see** the E, shake the screen 3 times and the App will instruct you to change the Testing Distance to **1m. Use your pre-cut 1m length of string.** Once the device is 1m from your child's face click 'done'

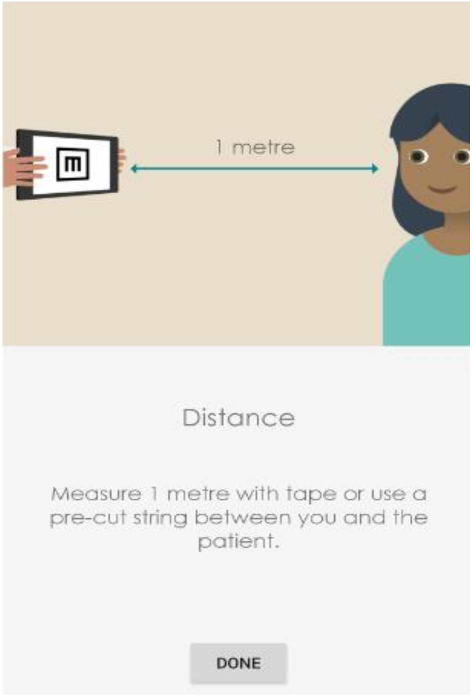

11. If your child is **able to see** the E at 1m instruct them to point in the direction they feel the E is pointing

12. If they are **unable to see** the E at 1m shake the device 3 times and the App will change the test distance to **30cm. Use your pre-cut 30cm length of string**

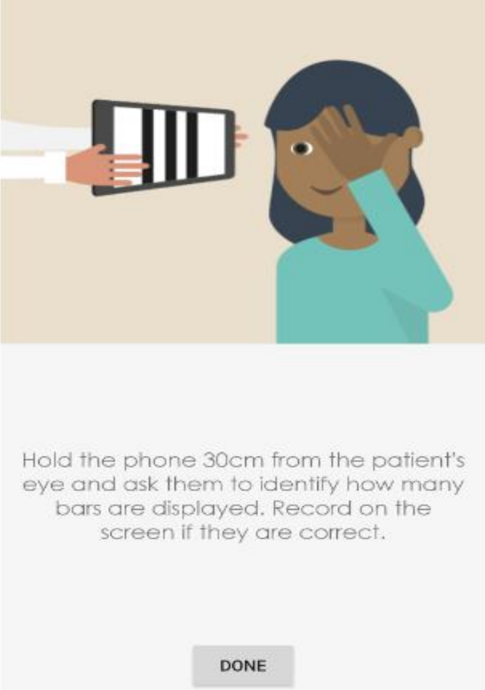

13. Your child will be presented with vertical strips – ask them to state whether they **Can't see** or **Can See** the stripes and then press the appropriate button

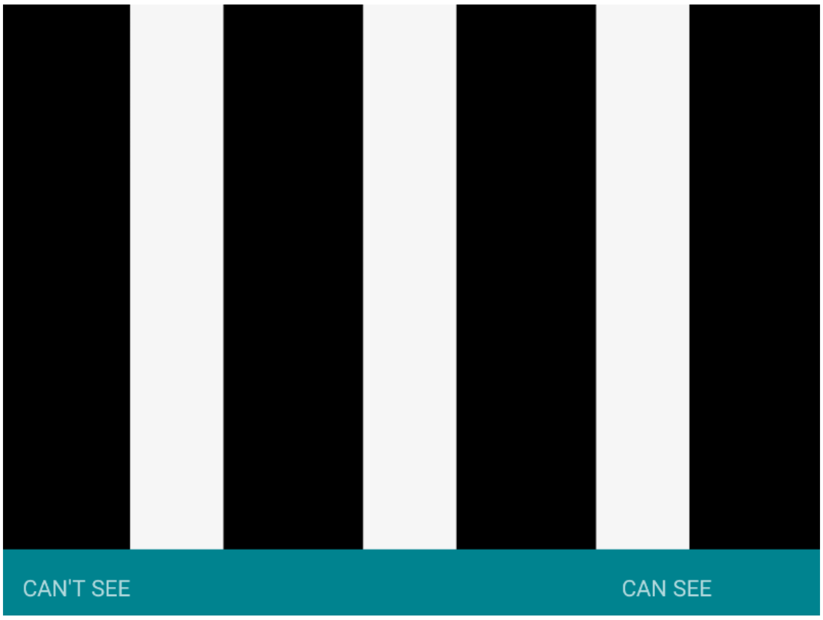

14. If your child **CAN'T SEE** the stripes they will then be presented with a moving black stripe – ask them whether they **Can't See** or **Can See** the moving stripe, then press the appropriate button

15. If your child **CAN'T SEE** the moving stripe the App will switch on the devices light, hold the light directly in front of the eye – ask the your child if they Can't See or Can See the light and then press the appropriate button

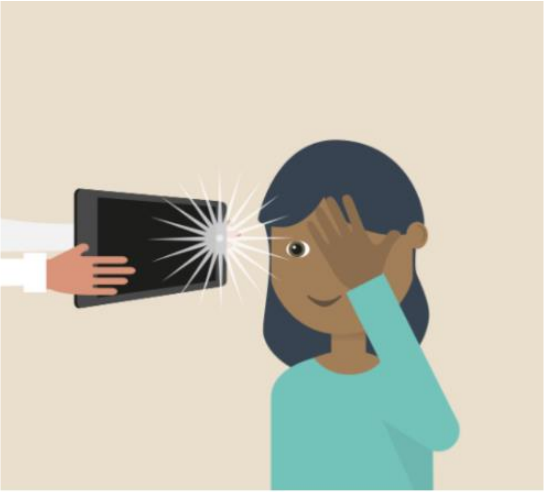

NOTE: Be careful to ensure the other eye is completely covered

16. At the end of the Vision Test you will be provided with a vision result

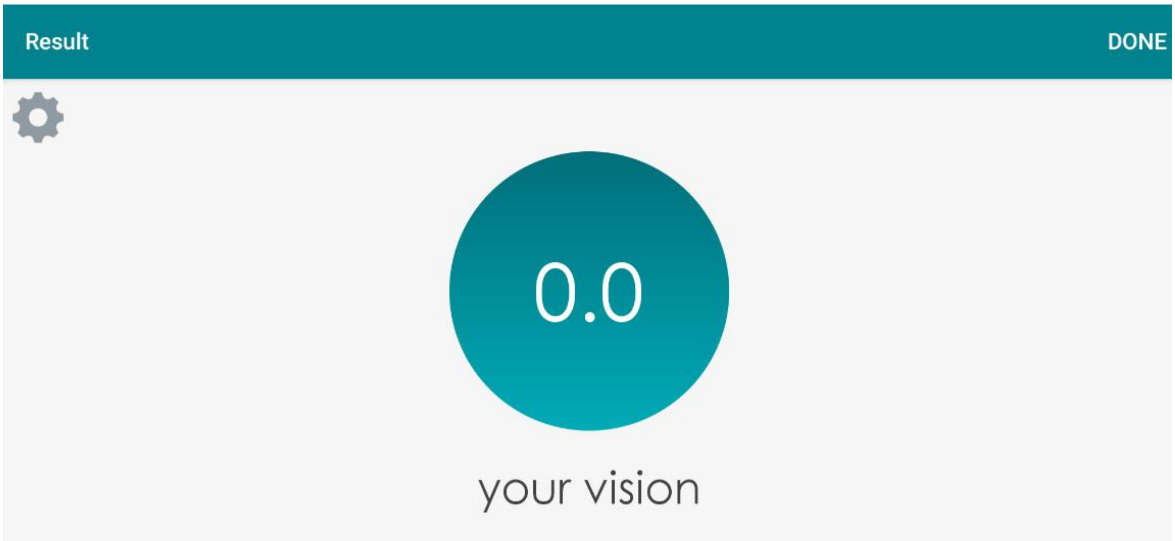

Be sure to document the number in the blue circle (your child's vision) for the Right and Left eye separately

17. Informing the Eye Department of your child's vision:

- Please inform your Orthoptist when they call you for your virtual appointment; if you have been requested to check your child's vision before the appointment.
- If your child's vision has been requested to help determine if they need to be seen in clinic, contact the Eye Department by Telephone: **0121 333 9467** or if you are unable to speak to someone email bwc.eyedepartment@nhs.net
