# Supplementary material for: Parental Home Vision Testing of Children During Covid-19 Pandemic
Source: Br Ir Orthopt J. 2021 Jan 21;17(1):13–9. doi: 10.22599/bioj.157 (PMC8269789; doi:10.22599/bioj.157)
Supplement: Appendix 2. — Parental questionnaire. [file bioj-17-1-157-s2.pdf]

Birmingham Women's and Children's
NHS Foundation Trust

Parental Questionnaire for the Home Vision Testing Apps

Thank you for taking the time to complete this questionnaire, your responses will help support the Eye Department evaluate the Home Vision Testing Apps usefulness as well as the Information Guides provided

My Child's name & Date of Birth___________________

My First Language is__________________

Which Vision Testing App did you use? **Please circle**	iSight (Apple device)	Peek Acuity Pro (Android device)
What device did you use? **Please circle**	Phone	Tablet

Please tick and rate how you found the Home Vision Testing Apps and the information guides

1 – Difficult **2** – No Problems **3** – Easy

	**1**	**2**	**3**
1. Finding the BCH vision testing instructions on the BCH Website was			
2. Understanding the vision testing instructions was			
3. I found testing my child's vision was			
4. I found measuring the appropriate distance was			
	**1**	**2**	**3**
5. My child found keeping their concentration for the duration of the test			
6. My child found covering one eye for the duration of the test			

Comments:
